# The origins of human pluripotent stem cells: the road from a cancer to regenerative medicine

**DOI:** 10.1007/s11626-024-00865-8

**Published:** 2024-02-23

**Authors:** Peter W. Andrews

**Affiliations:** The Centre for Stem Cell Biology, The School of Biosciences, The University of Sheffield, Western Bank, Sheffield, S10 2TN UK

**Keywords:** Embryonal carcinoma, Embryonic stem cells, Induced pluripotent stem cells, Pluripotent stem cells, Human, Mouse, Differentiation

## Abstract

The notion of using pluripotent stem cells (PSCs) as a source of differentiated cell types for replacement of disease or damaged tissues in regenerative medicine is now an active area of research, with approaches to treating eye diseases such as age-related macular degeneration or Parkinson’s disease now on the horizon. But the foundations for this research lie in a quite different area of science, namely the role of genetics of cancer. In this review, we trace the evolution of ideas starting with the discovery that strain 129 mice are particularly subject to develop germ cell tumors, through the identification of embryonal carcinoma (EC) cells as the stem cells of the teratocarcinoma manifestation of these tumors, to the recognition of their relationship to pluripotent cells of the early embryo, and eventually their role in the derivation of embryonic stem cells, first from mouse embryos and then from primates including humans. This is a story that illustrates how science commonly develops through the interests and insights of individual investigators, often with unexpected and unintended outcomes.

## Introduction

In 1954, Leroy Stevens was working at the Jackson Laboratory in Bar Harbor Maine, a research center established to use the laboratory mouse to investigate the origins of cancer (https://www.jax.org/news-and-insights/2014/november/85-years-of-discovery). Research there had shown that different cancers seemed to appear more often in some strains of mice than in others, suggesting a genetic link to cancer susceptibility. In that year, Stevens published a paper showing that the males of a mouse strain called 129 had a propensity to develop a testicular cancer known as a teratoma, which was unknown in other strains of mice (Stevens and Little 1954).

Teratomas are peculiar tumors that typically arise in the gonads and contain a wide array of jumbled tissues as if from an embryo that had become disorganized (Mostofi and Price 1973; Scully 1979). Although rare in humans, they had long attracted the attention of pathologists because of their unusual nature (Damjanov and Wewer-Albrechtsen 2013). In women, they are typically benign but they can grow to large sizes so are life threatening if not surgically removed. By contrast, in men, these tumors are almost always highly malignant and so were often designated teratocarcinomas. They tend to occur in young men after puberty when other cancers are rare, so in that age group they are among the most common cancers. The testicular tumors that Stevens found in 129 mice were also often malignant and could be maintained indefinitely by retransplantation to successive male 129 mice. They were therefore seen as a new tool for investigating this type of cancer.

Teratocarcinomas are distinguished from teratomas by the presence of a histologically distinctive, undifferentiated cell type called embryonal carcinoma (EC) (Damjanov and Andrews 2007) and it was thought that these are the cells that are responsible for the malignant properties of these tumors, as well as being able to differentiate into all the somatic cell types that characterize teratomas. A crucial early development of this cancer model was the demonstration by Larry Kleinsmith and Barry Pierce, published in 1964, that a single cell, likely an EC cell, isolated from a teratocarcinoma and transplanted to another mouse was able to generate another teratocarcinoma with a typical wide array of differentiated cell types (Kleinsmith and Pierce 1964). Work over the succeeding years confirmed the stem cell nature of EC cells. The sensitivity of these cells to new chemotherapeutic agents such as cis-platinum (Oosterhuis *et al*. 1984) also provided the basis for the current successful treatment of a type of cancer that had killed many young men in previous years (Einhorn 1981).

### Embryonal carcinoma cells and embryos

The complex histology of teratomas had always suggested a relationship to embryogenesis. Not only do they contain tissues derived from all three germ layers of the embryo—ectoderm, mesoderm, and endoderm—they often also contain structures known as embryoid bodies that morphologically resemble early embryos (Damjanov and Andrews 2016). In his extensive body of work following his 1954 publication, Stevens provided definitive evidence that teratomas in the mouse have a germ cell origin: in males they are initiated in utero from primordial germ cells around the time that they migrate into the genital ridge, and do not form in embryos homozygous for *Sl/Sl*, in which germ cells do not develop (Stevens 1967a, b). In humans, more circumstantial evidence indicated that here too testicular teratocarcinomas are initiated by a defect in germ cell development in utero, albeit that they typically do not manifest until after puberty (Skakkebaek *et al*. 1987). In females, it seems likely that ovarian teratomas may arise later following parthenogenetic activation of oocytes in the ovary (Stevens and Varnum 1974).

The germ cell origin of teratomas and teratocarcinomas helped to support the notion that their development reflects a caricature of embryogenesis. Further evidence was provided by the development of teratomas and teratocarcinomas from mouse embryos that had been transplanted to ectopic sites (Solter *et al*. 1970; Stevens 1970). The establishment and detailed characterization of mouse EC cell lines in vitro also contributed to understanding the relationship of teratomas to embryos. Brenda Finch and Boris Ephrussi were the first to successfully establish cell lines from explanted murine teratocarcinomas, and their cells retained the ability to differentiate when transplanted back into a mouse (Finch and Ephrussi 1967). Subsequently, several groups established such lines and showed that the cells could be cloned while retaining the capacity for differentiation in vitro and in vivo (Rosenthal *et al*. 1970; Martin and Evans 1974, 1975; Nicolas *et al*. 1975). Further, these pluripotent cells, now characterized as EC cells, were found to express markers, notably alkaline phosphatase (Bernstine *et al*. 1973) and a cell surface antigen named ‘F9’ (Artzt *et al*. 1973), in common with the pluripotent cells of the inner cell mass at the blastocyst stage of embryonic development. However, definitive proof of the embryonic character of mouse EC cells came from the direct demonstration, first by Ralph Brinster (Brinster 1974) and confirmed by others (Mintz and Illmensee 1975; Papaioannou et al. 1975), that EC cells injected into a mouse blastocyst, which was allowed to implant in a pseudo pregnant female, would take part in embryonic development and contribute to normal tissues of the mouse that was born.

These observations together with more detailed characterization of cultured mouse EC cells provided the basis for the discovery, independently by Martin Evans and Matt Kauffman (1981) and by Gail Martin (1981), that inner cell mass cells from mouse embryos, explanted to culture, could be maintained indefinitely in vitro, while maintaining pluripotency. Such embryo-derived cells, termed embryonic stem (ES) cells, proved capable of forming teratomas when transplanted to ectopic sites in adult mice, or taking part in development to form chimeric mice when transferred to blastocysts that were allowed to develop to term. Since the chimerism included the germline (Bradley *et al*. 1984), genetic manipulation of ES cells in culture provided a new route to generating ‘transgenic’ mice to investigate the function of key genes of interest in development or disease (Robertson *et al*. 1986).

### Human embryonal carcinoma cells

Building on the success of studies with mouse EC cells, during the 1970s, several researchers began to establish cell lines from human teratocarcinomas, from biopsies of testicular cancers. This work was driven in part by a medical interest in these tumors, but also by the thought that, as in the mouse, these cells might be useful tools for human embryology. Initially, the discovery that two such human teratocarcinoma cell lines contained cells expressing the F9 antigen, which had been used to characterize mouse EC cells, encouraged the view that human EC cells would closely resemble mouse EC cells (Hogan *et al*. 1977; Holden *et al*. 1977). However, in a detailed comparison of eight human teratocarcinoma cell lines, we found several that exhibited the typical morphology of mouse EC cells, and formed, in immunodeficient mice, xenograft tumors that were histologically similar to EC cells in clinical examples of human teratocarcinomas (Andrews *et al*. 1980). These cells did not express another, monoclonal antibody-defined, antigen, Stage-Specific Embryonic Antigen-1 (SSEA-1), which otherwise showed similar expression patterns to the F9 antigen, being also expressed by mouse EC cells and the ICM of mouse embryos (Solter and Knowles 1978).

In a subsequent study, we cloned and characterized in detail one particular human testicular teratocarcinoma cell line, 2102Ep (Andrews *et al*. 1982). These cells, which formed xenograft tumors that were recognizable by clinical histopathologists as pure embryonal carcinoma, likewise did not express SSEA-1 in culture provided that they were continually maintained at a high cell density. However, they did express another antigen, SSEA-3, that is expressed on cleavage stage mouse embryos but not on their inner cell mass cells, or mouse EC cells (Shevinsky *et al*. 1982). SSEA-3 had also been shown to mark EC cells in clinical testicular tumors (Damjanov *et al*. 1982). Nevertheless, if 2102Ep cells were cultured at low cell densities, they appeared to differentiate morphologically, apparently towards a trophoblastic lineage (Damjanov and Andrews 1983), when they did begin to express SSEA-1 while downregulating SSEA-3. Thus, we concluded that human EC cells differ from their murine counterparts, at least with regard to expression of these cell surface antigens, and that in humans, in contrast to mice, EC cells, expression of SSEA-1 is an indicator of differentiation.

Unfortunately, 2102Ep cells and several of the other human teratocarcinoma cell lines initially available showed little sign of further differentiation into clearly identifiable somatic cells. Subsequently, we and others were able to identify human EC cell lines that did show somatic differentiation and did exhibit the antigen phenotype that we first characterized in 2102Ep cells (Andrews *et al*. 1984b; Thompson *et al*. 1984; Pera et al. 1989). In particular, we studied in more detail a pluripotent human EC cell line, NTERA2, which formed well-differentiated xenograft teratocarcinomas in immunosuppressed mice, and differentiated extensively in vitro, generating neurons as well as other cell types, in response to retinoic acid (Andrews *et al*. 1984b; Andrews 1984). Using these cells, additional developmentally regulated cell surface antigens of human EC cells were characterized, including SSEA-4, TRA-1–60 and TRA-1–81, and GCTM2 and differences from mouse EC cells were confirmed (Kannagi *et al*. 1983; Andrews *et al*. 1984a; Fenderson *et al*. 1987; Pera *et al*. 1988). What was unclear at this stage was whether the differences reflected species differences or differences in embryonic stage to which the cells correspond.

### Human embryonic stem cells

Unlike mouse EC cells, which are typically diploid though occasionally with some limited chromosomal rearrangements, human EC cells are generally highly aneuploidy, typically with an approximately triploid chromosome number with many rearrangements. Further, even the best human EC cells seemed limited with respect to the differentiated cells they would form. Consequently, from the early days following the description of mouse ES cells, there was an interest in whether it would be possible to derive corresponding cells from human embryos. In principle, human embryos could be obtained after the successful development of in vitro fertilization, but progress was hampered not only by the logistical problems of accessing embryos, but also by ethical concerns about the use of human embryos in research.

A significant step forward came from the derivation of ES cells first from rhesus monkey and then marmoset embryos by Jamie Thomson, working at the Wisconsin Primate Centre (Thomson *et al*. 1995, 1996). Strikingly, these monkey ES cells more closely resembled human rather mouse EC cells with respect to their surface antigen phenotype. Nevertheless, they were capable of extensive differentiation in vitro and formed well-differentiated teratomas in xenogeneic hosts. Importantly, they provided the experience for Thomson to derive ES cell lines from human embryos some 3 yr later (Thomson *et al*. 1998). Again, these human cells also closely resembled human EC cells, but with normal karyotypes and a capacity for extensive differentiation.

### Opportunities and challenges

Following the first publication describing human ES cells, the notion that these cells could provide a source of differentiated cells to replace diseased or damaged tissues, a field now often encapsulated by the term ‘regenerative medicine,’ gained rapid traction (Gearhart 1998; Pedersen 1999; Daley 2002). In fact, the idea had already been floated by a group working with neuronal derivatives of NTERA2 EC cells for the treatment of stroke (Borlongan *et al*. 1998; Kondziolka *et al*. 2000), but ES cells with their apparently normal karyotype and extensive capacity for differentiation were immediately seen as better candidates for the approach. Also, reversing the extensive damage caused by stroke presents enormous challenges making it a poor candidate for early trials. Interest then coalesced around medical conditions that were confined to the well-characterized loss of particular cell types, notably diabetes, age-related macular degeneration (AMD), and Parkinson’s disease. Despite the many challenges of preparing cells to standards that permit regulatory approval for clinical applications, trials of transplanting retinal pigment cells derived from ES cells for treating AMD begun within 20 yr of the first description of human ES cells (Schwartz *et al*. 2012; Vitillo *et al*. 2019; da Cruz et al. 2018), and trials for Parkinson’s disease have also been planned (Barker *et al*. 2017). These two conditions have the further advantages for first in man trials because they affect cells in confined organs, the eye and the brain, that may represent immune privileged sites, they required only small numbers of cells, and there were already prior studies that provide a proof of concept that the approach could potentially effect a cure.

In parallel with the interest in regenerative medicine, it also became apparent that the differentiation of ES cells could offer opportunities to obtain large numbers of differentiated cell types that could be used for testing the safety and efficacy of potential new drugs, or for exploring the mechanisms of many medical conditions. Indeed, in the pharmaceutical industry, many candidate drugs fail in late stages of development because of liver or cardiac toxicity. Consequently, much effort has been put into generating hepatocytes and cardiomyocytes from ES cells for this purpose (Lu and Yang 2011; Meseguer-Ripolles *et al*. 2018). Likewise, recent studies have developed ways to produce ‘embryoids’ from ES cells to provide tools for investigating early embryogenesis and causes of abnormal development (Amadei *et al*. 2022).

However, notwithstanding the great excitement about the potential uses of ES cells, exploiting their potential has also faced many challenges. Of these, perhaps the biggest has been the ethical issues of research involving human embryos (https://www.eurostemcell.org/embryonic-stem-cell-research-ethical-dilemma). To some, any experimental work with human embryos is an anathema, and this is reflected in the laws and regulations preventing work with ES cells in some jurisdictions. To others, for example in the UK, a more pragmatic approach is acceptable, so that work with early embryos, typically up to 14 d post fertilization, is legally permissible for particular purposes such as the production of ES cells. However, the discovery by Shinya Yamanaka and others (Takahashi and Yamanaka 2006; Takahashi *et al*. 2007; Yu *et al*. 2007) that somatic cells can be reprogrammed to a state similar, if not identical to that of embryo-derived ES cells, has provided a solution to this problem. These so-called induced pluripotent stem (iPS) cells typically express all the characteristics of ES cells including their capacity for differentiation, though present their own challenges such as the need to avoid genetic changes resulting from retention of the genes transfected into the cells to achieve reprogramming. Nevertheless, iPS cells are now a widely used alternative to ES cells in many studies, and trials of iPS cell–derived derivatives for AMD and Parkinson’s disease have been initiated (Takahashi 2021; Akiba et al. 2023).

Of the other practical difficulties in exploiting the opportunities of ES and iPS cells, perhaps the most tricky is their propensity to acquire genetic changes after prolonged passage (Halliwell *et al*. 2020a). Although human ES and iPS cells are typically euploid when first derived, they have a propensity to acquire non-random karyotypic changes, particularly gains of the long arm of chromosomes 1, 17, and 20, and the short arm of chromosome 12. Intriguingly, these changes are also common among the many other deviations from diploidy in human EC cells. This propensity for non-random karyotypic change has been since widely confirmed (The International Stem Cell Initiative *et al*. 2011; Andrews *et al*. 2017). In addition to karyotypic changes, smaller genomic changes including single base changes also occur and these may also be non-random such as variants affect *TP53* (Merkle *et al*. 2017). These non-random variants can appear in cultures very rapidly and almost certainly reflect selective growth advantages that they confer on the cells.

In contrast to somatic cells, ES and iPS cells are particularly susceptible to DNA replication stress and the formation of double strand breaks (Halliwell *et al*. 2020b). Surprisingly, however, the overall mutation rate in ES cells is very low, comparable to that of somatic cells and much lower than many cancer cells (Thompson *et al*. 2020). These seemingly contradictory observations can be reconciled by a further observation that in response to DNA replication stress of ES and iPS cells tend to die through apoptosis, in contrast to somatic cells (Desmarais *et al*. 2012, 2016). Tellingly, many of the non-random changes seen in ES and iPS cells appear to control apoptosis; e.g., gains of the long arm of chromosome 20 appear to be driven by increased expression of *BCL2L1* located on that chromosome (Avery *et al*. 2013). Cells with such mutations appear able to escape apoptosis in response to DNA damage (Halliwell *et al*. 2020a).

Although progress is being made in understanding the mechanisms by which genetic variants arise, assessing the consequences of particular genetic variants for different applications remains a substantial problem. A recent report on ‘Standards for Research with Human Stem Cells’ by the International Society for Stem Cell Research (https://www.isscr.org/standards) highlighted this point, and strongly recommended that careful attention is paid to reporting in full the nature of any genetic variants present in cells used for particular experiments, so that retrospective analysis may provide important clues in the future. The biggest concern is the possibility of cancer developing in patients arising from derivative cells used for regenerative medicine applications. Although animal models of tumorigenicity can be useful, they are expensive to carry out and certainly cannot provide a definitive conclusion as the tumorigenic potential of particular variant cells in specific human situations. It is likely that new approaches, perhaps based on extensive bioinformatics data, will be needed.

## Conclusion

It is now 70 yr since Leroy Stevens described the susceptibility of strain 129 mice to testicular teratomas. In that period, experimental research with these tumors and cell lines derived from them has laid the foundations for the development of ES cells, and eventually iPS cells in both mouse and humans. ES cells from the laboratory mouse continue to provide the chief means for genetically manipulating mice to provide tools to address the mechanisms of embryonic development and the causes of abnormal fetal development, as well as the causes of aging and disease in the adult. Despite the continuing challenges of controlling their differentiation to specific cell types and addressing the problems of culture-induced genetic variation, human ES and iPS cells now offer important opportunities for regenerative medicine as well as for optimizing drug discovery and understanding disease mechanisms in humans. It is striking, though, that these opportunities were not obvious when the first mouse teratocarcinoma lines were isolated.
